# Human Serum Albumin and p53-Activating Peptide Fusion Protein Is Able to Promote Apoptosis and Deliver Fatty Acid-Modified Molecules

**DOI:** 10.1371/journal.pone.0080926

**Published:** 2013-11-21

**Authors:** Michelle R. Joshi, Nianhuan Yao, Kenneth A. Myers, Zhiyu Li

**Affiliations:** 1 Department of Pharmaceutical Sciences, Philadelphia College of Pharmacy, University of the Sciences, Philadelphia, Pennsylvania, United States of America; 2 Department of Biological Sciences, Misher College of Arts and Sciences, University of the Sciences, Philadelphia, Pennsylvania, United States of America; Aberystwyth University, United Kingdom

## Abstract

Therapeutic peptides offer a high degree of specificity, potency, and low toxicity; making them promising candidates for cancer therapy. Despite these advantages, a number of hurdles, such as poor serum stability and inefficient cellular penetration, must be overcome. Fusing a therapeutic peptide to human serum albumin (HSA) is a common approach to extend the serum stability of a peptide that binds to extracellular receptors. However, no study has shown that this approach can be applied to target intracellular proteins. Here we demonstrate the feasibility of using a recombinant human serum albumin (rHSA) fusion protein to simultaneously deliver two types of molecules: a peptide capable of binding an intracellular target, as well as fatty acid (FA)-modified FITC (FA-FITC). Two peptides reported to disrupt the intracellular p53 and MDM2/MDMX interaction were fused to the C-terminal of HSA. Cellular and biochemical studies indicate that rHSA fusion proteins were efficiently taken up by SJSA-1 cells and retained MDM2- and MDMX-binding activity. By inducing the accumulation of p53, both fusion proteins promoted efficient cytotoxicity in SJSA-1 cells via caspase activation. Long chain fatty acid (LCFA) transportation is an essential endogenous function of HSA. This study also demonstrates that rHSA fusion proteins formed highly stable complexes with FA-FITC via non-covalent interactions. FA-FITC complexed with HSA could be internalized efficiently and rHSA-P53i and rHSA-PMI retained apoptotic activity as complex components. It is expected that such an approach can ultimately be used to facilitate intracellular delivery of two anticancer therapeutics, each with distinct but complimentary mechanisms, to achieve synergistic efficacy.

## Introduction

Use of proteins and peptides as therapeutic agents has become increasingly provocative in recent years. These biologically active molecules have many advantages over small molecule drugs including higher specificity and decreased potential to cause adverse effects. The average number of new peptide drug candidates has grown steadily from an average of 1.2 per year in the 1970’s to 16.8 per year so far in the 2000’s [1]. Among these promising candidates, however, few are known to bind intracellular proteins, thus ignoring a vast reservoir of potential targets. An efficient cell penetrating technology remains one of the major obstacles to peptide drug application. Here we propose a delivery technology using recombinant human serum albumin (rHSA) to promote cellular penetration of both a therapeutic peptide and small molecule drug. In addition to prolonging serum stability, this novel strategy is capable of facilitating simultaneous intracellular delivery of two therapeutic agents, each with distinct but complimentary mechanisms, to promote a synergistic therapeutic response for the treatment of a variety of diseases.

Our initial model to test this delivery technology uses a p53-derived peptide to target the anti-apoptotic interaction between two intracellular proteins, p53 and MDM2. The p53 tumor suppressor protein plays a critical role in generating cellular responses to a number of stress signals, including DNA damage, aberrant proliferative signals due to oncogene activation, and hypoxia. Upon activation, p53 is stabilized and moves to the nucleus, where it binds to DNA in a sequence-specific manner and promotes transcriptional regulation of genes involved in DNA repair, cell-cycle arrest, senescence, and apoptosis [2,3]. Previous studies have demonstrated that p53-mediated apoptosis plays a critical role to suppress tumor formation in mice [4]. While it is estimated that the p53 gene is mutated in 50% of tumors, increasing evidence reveals that a large percentage of tumors retain wild type p53, but possess other alterations in the p53 pathway, which prevent its critical tumor-suppressive function [5]. One key component altering p53 activity is the E3 ubiquitin ligase, MDM2. This negative regulator directly binds to p53 and promotes the ubiquitination and subsequent proteasomal degradation of p53. Under normal conditions, MDM2 functions as a harness for p53 activity, regulating its subcellular location, transcriptional activity, and stability. In tumors, however, MDM2 is frequently upregulated, thus preventing the p53 stress response even in cases where wild type p53 is present. As a result, patients often display accelerated tumor growth and a diminished response to treatment [6,7]. Disruption of the p53-MDM2 interaction has become a popular strategy to increase functional p53 levels and thus, reduce cancer cell viability.

The binding interface of p53-MDM2 is composed of a hydrophobic cleft on the N-terminal surface of MDM2 and the N-terminal transactivation domain of p53. Since the revealing of the interaction interface, a series of small molecules and peptides have been developed to target the p53-binding pocket of MDM2 [8]. One such class of small molecule antagonists termed nutlins, have demonstrated the ability to dock within the p53-binding pocket of MDM2, resulting in p53 accumulation, initiation of cell cycle arrest, and ultimately, apoptosis [9,10]. Despite this, translation into an effective treatment modality has shown little promise. The limited effect of small molecule p53-MDM2 inhibitors is thought to be in part due to the lack of inhibition of MDMX, a homolog of MDM2 [11,12]. In depth analyses of MDM2 and MDMX revealed both proteins work in concert to decimate the p53 pathway, thus necessitating the development of an inhibitor with dual specificity [13]. Rationally designed synthetic peptides offered an alternative to nutlins and other small molecule antagonists by binding and inhibiting both MDM2 and MDMX. PMI peptide was developed by Li and colleagues to compete with p53 for MDM2 and MDMX binding at an affinity approximately 2 orders of magnitude higher than that of a wild type p53-derived peptide (containing amino acids 17-28 of the p53 protein) [14,15]. While this elegant work gave considerable support to the concept of targeting the p53-MDM2/MDMX interaction for cancer therapy, problems surrounding proteolytic stability and intracellular peptide delivery still remained. 

Interest in using HSA as a drug carrier has grown in recent years due to a number of properties including: preferential uptake in tumor and inflamed tissue, stability, biodegradability, ready availability, and lack of toxicity and immunogenicity [16–18]. HSA is capable of improving the pharmacokinetic profile of peptide- or protein-based drugs by chemical conjugation, genetic fusion, and micro/nano particle encapsulation [19]. HSA is the most abundant plasma protein with an average half-life of 19 days. It functions as a natural transport vehicle for metal ions, a number of drugs, and long chain fatty acids in the blood [20]. In addition, tumor cells often have an increased rate of albumin uptake. For example, HSA makes up 19% of the soluble proteins within certain breast cancer cells [21]. A number of drugs have been designed to exploit these valuable characteristics of HSA. Acylated insulin and glucagon-like peptide-1, which rely on HSA-mediated binding to extend serum stability, have been approved for clinical use [22,23]. The HSA/paclitaxel nanoparticle known as Abraxane was approved for the treatment of metastatic breast cancer and has shown promise as a delivery strategy to extend the half-life and therapeutic efficacy of small molecule drugs [19]. A major concern of albumin-based formulations such as Abraxane, however, is the uncertainty surrounding their aptitude for generating an immune response against endogenous HSA. Based on extensive *in vivo* studies and several clinical trials, no such immune response has been reported, even for HSA fused to immunostimulating cytokines, such as interferon α2b (Albuferon) [24]. Furthermore, Recombumin, a genetically engineered form of HSA used to replace endogenous albumin, is already in therapeutic use and has a proven lack of toxicity and immunogenicity [17]. 

The novel technology presented in this paper utilizes the long-chain fatty acid transportation properties of HSA with its aptitude as a genetically modifiable protein to deliver a highly specific peptide to an intracellular target. Acylation with long chain FA is a method that has previously been used to extend the serum half-life of small compounds by facilitating non-specific association with serum albumin and lipoproteins [25]. In contrast to the acylated drugs currently approved for clinical uses that rely on random serum protein association *in vivo*, our HSA-mediated delivery technology is pre-formulated under optimized *in vitro* conditions to guarantee simultaneous intracellular delivery of two complimentary therapeutic agents. This strategy allows drug transportation and release to mimic the robust fatty acid uptake as well as albumin transportation. 

Combination therapy is a common approach for cancer treatment. Delivery of multiple therapeutics, simultaneously to one target, can improve efficacy and minimize toxicity. Encapsulated micro/nano particles and conjugated polymers have been developed to co-deliver different therapeutics. However, they are plagued by a number of factors including immunogenicity, difficulty in penetrating solid tumors, lack of selectivity for target tumor tissue, inefficient dissociation from a covalently-bound carrier, and reliance on passive diffusion, a process that does not guarantee co-delivery of both anticancer agents to the same cell [26,27]. Our data suggest HSA is a feasible choice to serve as a protein carrier for co-delivery of C-terminal fused peptides and FA-modified molecules. In the realm of cancer treatment, it is our hope that such a system can ultimately be used to facilitate intracellular delivery of two anticancer agents, each with distinct roles in regard to triggering or responding to cellular DNA damage, to promote a more robust apoptotic response for the treatment of solid tumors. 

The purpose of this paper is to demonstrate the feasibility of using genetically modified HSA to fuse a therapeutic peptide, while retaining FA-binding ability for its eventual use as a carrier to co-deliver both a peptide and FA-modified Drug (FA-Drug). Two fusion HSA proteins containing either a wild type p53-derived peptide (P53i) or the high affinity MDM2-binding peptide N8A-PMI (PMI) described above were cloned, expressed in *Pichia pastoris* yeast system, and purified [28]. Cellular and biochemical studies indicate that rHSA-P53i and rHSA-PMI were efficiently taken up by osteosarcoma SJSA-1 cells and retained MDM2- and MDMX-binding activity. In addition, both rHSA-P53i and rHSA-PMI promoted cytotoxicity in SJSA-1 cells via caspase activation. As the future application of this rHSA delivery technology aims to deliver one or more FA-Drugs in addition to a C-terminal-fused therapeutic peptide, FA-binding and stability studies were also performed using FA-FITC. As expected, engineered rHSA proteins (rHSA-P53i and rHSA-PMI) were able to form highly stable complexes with FA-FITC via non-covalent interactions. FA-FITC complexed with HSA could be internalized by the target cells and rHSA fusion proteins still retained cytotoxicity. 

## Methods

### Design and Expression of rHSA-p53 and rHSA-PMI

Recombinant HSA fusion proteins were cloned into pPICZαA vector (*Invitrogen*). The 5’ primer contained a 21 base pair sequence overlapping with the N-terminal of HSA cDNA and the XhoI cloning site of the vector (5’-ATCGCTCGAGAAAAGAGAGGCTAAGCGACGCACACA AGAGTGAGGTTGCT-3’). The 3’ primer contained a portion of the C-terminal of HSA, wild type P53i (ETFSDLWKLLPE) or PMI (TSFAEYWALLSP) peptides, as well as the NheI restriction site. This sequence was subsequently amplified by PCR. Each peptide sequence was fused to the C-terminal of HSA by overlapping PCR with primers: 5’-CCATAGGTCTGAAAACGTTTCACCTCAACTTCGTCGGCGCCTAAGGCAGC-TTGACTTGCAGC-3’ (HSA C-terminal), 5’-CGATGCTAGCAC TAGTTTATTCA-GGAAGTAGTTTCCATAGGTCTGAAAACGTTTCACC-3’ (rHSA -P53i, C-terminal), 5’-CGATGCTAGCCCGCGGTTATGGACTAAGAAGAGCCCAGTACTCAGCAAA-ACTTGTACCGTCAACTTCGTCGGCGCC-3’ (rHSA-PMI, C-terminal). The pPICZαA vector was digested with XhoI and XbaI to create the 5’ and 3’ cloning sites. The HSA fusion protein sequences were digested with XhoI and NheI and ligated into the linearized vector. Following ligation and transformation, the cloned genes were confirmed by DNA sequencing. *Pichia pastoris* yeast cells (*Invitrogen, 18258-012*) were then transformed using linearized pMM1 (for HSA-P53i) or pMM2 (for rHSA-PMI) plasmid DNA and rHSA-P53i and rHSA-PMI clones were selected for Zeocin resistance after 72 hours. Recombinant proteins were then expressed in *Pichia pastoris* according to the manufacturer’s instructions (*Invitrogen, K1740-01*). 

### Purification of rHSA-P53i and rHSA-PMI

Culture media containing albumins secreted from *P. pastoris* were filtered (0.2 µm) and subsequently incubated for 4 hours at 4°C with cibacron blue dye agarose (Sigma, C9534) [29]. Following 10X volume washes with ice-cold PBS, recombinant proteins were eluted stepwise using PBS containing sodium thiocyanate (NaSCN) (100 mM, 200 mM, 500 mM, 750 mM and 1 M). Fractions containing rHSA-P53i or rHSA-PMI (500 mM to 750 mM NaSCN) were pooled and dialyzed in PBS. Purified proteins were analyzed by SDS-PAGE with purity greater than 95%. Recombinant HSA proteins were then filtered (0.2 µm) for sterilization and stored at -20°C. Protein concentration was determined using the Bradford method (*Bio-Rad, 23225*). 

### FITC and biotin-labeling of rHSA

FITC and biotin-labeling of rHSA (biotin-rHSA) were performed using NHS-Fluorescein (*Thermo Scientific, 46409*) and NHS-Biotin (*Thermo Scientific, 20217*) according to the manufacturer’s instructions. Both labeling procedures were performed using 20 times excess of NHS. Following the incubation, proteins were dialyzed in 1X PBS to remove unconjugated NHS reagents.

### Cell Culture and Cytotoxicity Studies

Osteosarcoma SJSA-1 cells (*ATCC*) were grown in RPMI media containing 10% FBS. Cytotoxicity assays were performed using SJSA-1 cells plated in 24- or 96-well plates at 20,000 or 5,000 cells per well, respectively. Cells were allowed to attach overnight. All treatments were added on day 2 in RPMI media containing 1% FBS plus the equivalent amounts of 1X PBS buffer. Unless otherwise indicated, cells were exposed to treatment media for 24 hours, at which time cytotoxicity was measured using the fluorometric *CyQuant* assay (*Invitrogen, C35006*) or the fluorometric *Homogeneous Caspase* assay (*Roche, 03005372001*), according to the manufacturer’s instructions, for detection of apoptosis. All results were plotted relative to rHSA- or where indicated, rHSA/FA-FITC-treated cells. 

### Confocal Microscopy

On day 1, SJSA-1 cells were seeded in 6-well plates, at a density of 80,000 cells per well, and allowed to attach overnight. Treatment media containing 1% FBS plus 5 µM FITC-labeled rHSA (FITC-rHSA), FITC-rHSA-P53i or FITC-rHSA-PMI was added to wells on day 2 and allowed to incubate for 24 hours. For experiments to examine the internalization activity of FA-FITC-modified rHSA, SJSA-1 cells were plated as described above. On day 2, rHSA fusion proteins (dissolved in 1X PBS) were incubated with FA-FITC at a 1:2 molar ratio (rHSA:FA-FITC; 5 μM:10 μM) to allow formation of rHSA/FA-FITC complexes. Reactions were conducted in PBS at room temperature for 30 minutes, prior to dilution in RPMI media (without FBS) and addition to wells. Following a 24 hour incubation period, cells were trypsinized, re-plated onto coverslips and allowed to re-attach for 2 hours. Upon re-attachment, cells were washed 3X with PBS and maintained in phenol red-free media. For visualization, imaging was performed using a Nikon TiE (*Eclipse*) confocal microscope with a CSU-X spinning disk confocal scan head (*Yokogawa*), a linear encoded x, y robotic stage (*ASI Technologies, Inc.*), equipped with a multi-bandpass dichromatic mirror and bandpass filters (*Chroma Technology Corp.*) in an electronic filter wheel for selection of FITC.  488 nm laser illumination was provided by a 50 mW monolithic laser combiner (*MLC400, Agilent Technologies*) and images were acquired using a 60x 1.40NA objective and the Clara interline CCD camera (*Andor Technology*).

### Co-Immunoprecipitation and Protein Detection by Western Blotting

SJSA-1 cells were lysed in buffer containing 20 mM Tris-HCl, 50 mM NaCl, 0.05% Triton X-100, and protease inhibitor cocktail. For each condition, 200 µg SJSA-1 cell extract was heated to 42°C prior to the addition of 4 µg biotinylated rHSA-P53i, rHSA-PMI or rHSA protein. Mixtures were then allowed to incubate for 1 hour at RT. Next, MDM2 (*Santa Cruz, sc-965*) or MDMX antibody (*Santa Cruz, sc-74467*) was added to each tube and allowed to incubate, while rotating, for 4 hours at 4°C. Proteins bound to MDM2/MDMX antibody were pulled down using Protein A/G (1:1) resins and samples were analyzed by SDS-PAGE and Western blotting using MDM2, MDMX, and Streptavidin-HRP antibodies (*Pierce, 21130*). Primary and secondary antibodies were added in 2% non-fat dry milk in TBST at the following dilution ratios: p53 (*Santa Cruz, sc-126*), MDM2, MDMX (1:200), streptavidin-HRP (1:2500), and GAPDH (1:5000) (*Santa Cruz, sc-59541*). Proteins were visualized by the chemiluminescent detection solution, SuperSignal West Dura Extended Duration Substrate (*Thermoscientific, 34075*) and densitometry was performed on replicate experiments using Image J software (*NIH*).

### Gel shift assays to determine stability of FA/HSA complexes

All recombinant albumins were defatted following a previous publication [30]. FA-FITC was synthesized by mixing 1X 1-Hexadecylamine and 2X N,N-Diisopropylethylamine (*Sigma*) followed by addition of 1X NHS-Fluorescein (*Thermo Scientific*). This reaction was carried out overnight, protected from light. Products were then purified by HPLC and identified by MS. For experiments designed to detect FA/HSA complex formation, rHSA (30 pmol, dissolved in 1X PBS) was incubated with or without FA-FITC at desired molar ratios and then separated by 0.5X TBS PAGE. Due to the incorporation of fluorescent molecules, HSA bound by FA-FITC in gel can be visualized under UV. Assays to determine the degree of displacement of FA-FITC were performed by adding an excess amount of unlabeled FA to pre-formed rHSA/FA-FITC complexes, at the indicated molar ratios. Reactions were conducted in PBS containing 10% glycerol at room temperature for 30 minutes. The products were separated using non-denaturing 0.5X TBS PAGE and then visualized under 305 nm UV. Experiments to determine the effect of the presence of serum on HSA/FA complexes were conducted using pre-formed biotin-rHSA/FA-FITC complexes. After initial complex formation, the biotin-rHSA/FA-FITC solution was divided equally among 4 tubes. Ten percent FBS or the same volume of PBS was subsequently added to respective samples and the mixtures were allowed to incubate for up to 24 hours at 37°C followed by the addition of streptavidin-conjugated resins to pull-down biotinylated rHSA/FA-FITC complexes (*GenScript, L00353*). Streptavidin resins were pre-equilibrated with HSA to minimize nonspecific interactions. Samples performed in parallel to assess total FA-FITC incorporation did not receive streptavidin resins, but underwent an identical incubation time of either 1 or 24 hours at 37°C. Next, samples were centrifuged to pull down biotin-HSA/FA-FITC and a portion of each supernatant was analyzed using 0.5 X TBS PAGE as above. 

## Results

### HSA fusion protein design, expression, and purification

The structural basis for MDM2/MDMX interaction with p53 is well understood. To form a complex with MDM2 or MDMX, the amphipathic α-helix fragment of the N-terminal transactivation domain of p53 must bind within the concave binding pocket of MDM2/MDMX. Despite minor sequence differences and a slightly smaller hydrophobic binding cleft in MDMX compared to MDM2, structural studies reveal that the p53 binding domains of both proteins display a high degree of similarity. The minimally required MDM2/MDMX binding sequence includes residues 19-26 of the transactivation domain of wild type p53 (**F** ^19^S^20^D^21^L^22^ **W** ^23^K^24^L^25^ **L** ^26^). Noteworthy are three critical residues, known as the hydrophobic triad (F^19^W^23^L^26^), which bind to the three distinct sites of the MDM2/MDMX hydrophobic pocket [8,31,32]. 

**Figure 1:**
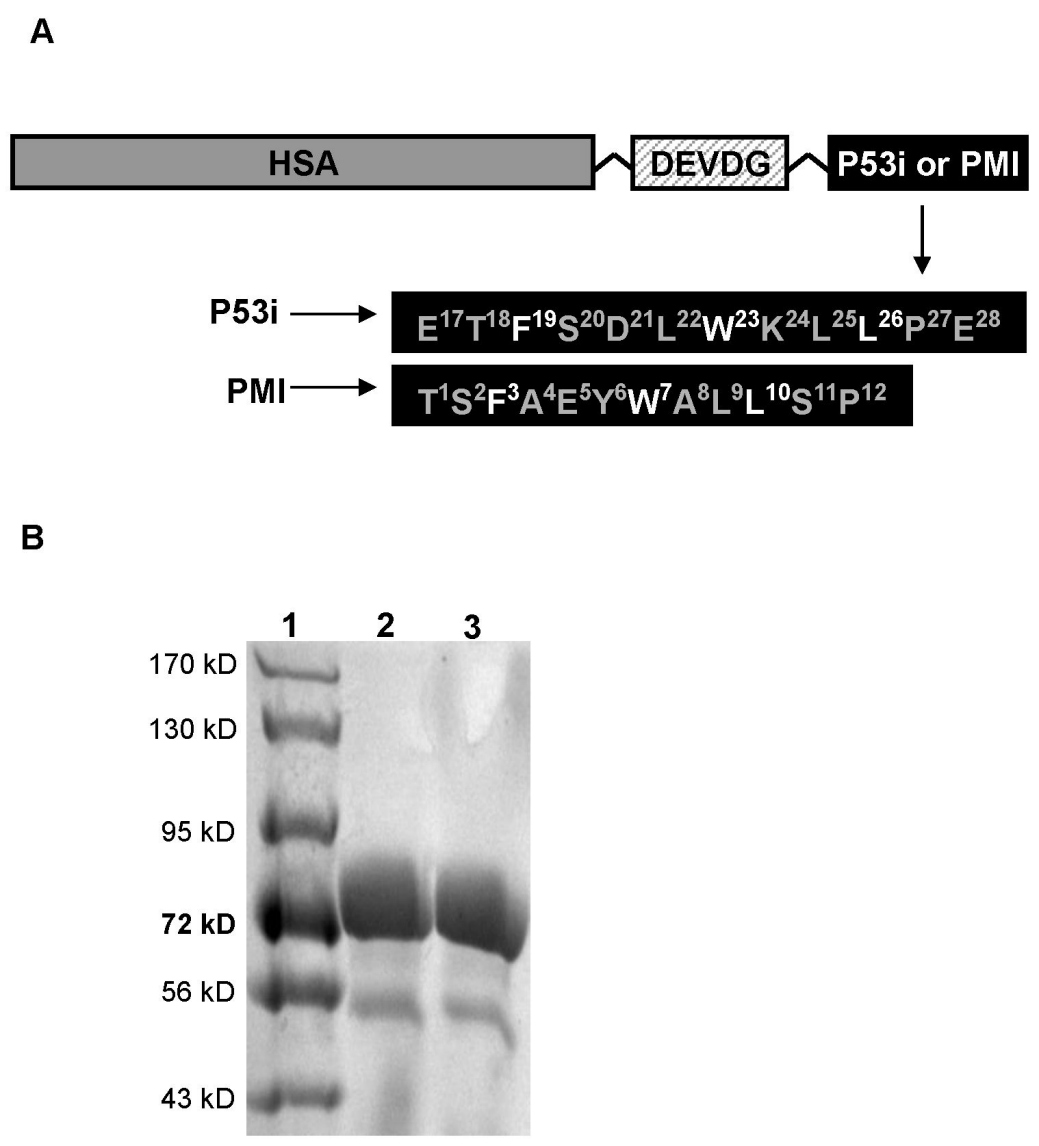
Design and purification of rHSA-P53i and rHSA-PMI. A). rHSA/peptide fusion protein was constructed by fusing either P53i or PMI peptide sequence to the C-terminal of HSA. A caspase cleavage site (DEVDG) was included as a linker between HSA and peptide. B). Fusion proteins were expressed using *Pichia pastoris* yeast expression system and purified by cibacron blue dye agarose as described in *Methods*. Lane 1 contains 10 µl of pre-stained *Rec* protein ladder (Fischer). Lanes 2 and 3 contain 10 µg of purified rHSA-p53i and rHSA-PMI, respectively. Proteins were visualized by coomassie blue staining, revealing >95% purity and MW of approximately 70 kD.

In this study, two peptide sequences were chosen to test our initial HSA-mediated peptide delivery approach. The first construct fused to HSA contains the wild type p53 binding sequence (E^17^T^18^ **F** ^19^S^20^D^21^L^22^ **W** ^23^K^24^L^25^ **L** ^26^P^27^E^28^) and the second sequence is a potent MDM2/MDMX peptide inhibitor, PMI (TS**F**AEY**W**NL**L**SP), adopted from the work of Li and colleagues [14,15]. To avoid the effect of bulky HSA structure on peptide-MDM2/MDMX interaction, a caspase cleavage site (DEVDG) was inserted as a linker between HSA and peptide (Figure 1A). The insertion of this linker may facilitate liberation of peptides from HSA following p53 accumulation and subsequent caspase activation. 

The wild type p53-derived peptide (P53i) or PMI peptide sequences were fused to the C-terminal of HSA (Figure 1A) using a protocol as described in *Methods*. The fusion proteins were then cloned into pPICZαA *Pichia pastoris* protein expression vectors and transformed into yeast cells. Recombinant HSA-P53i and rHSA-PMI were overexpressed and purified using cibacron blue dye agarose to achieve >95% purity confirmed by SDS-PAGE (Figure 1B). 

### Both rHSA-P53i and rHSA-PMI are efficiently internalized by SJSA-1 cells

Therapeutic activity hinges upon successful delivery of a peptide or small molecule drug into the cell. While new strategies are consistently being evaluated to deliver functional proteins or peptides into cells, they are still lacking in overall efficiency and safety for translation into a clinical model [1]. Current HSA drug formulations such as Abraxane, the HSA-paclitaxel nanoparticle, demonstrate efficient intracellular HSA uptake. Multiple modes of internalization have been shown to play a role, including receptor-mediated as well as endocytic pathways [19]. 

**Figure 2:**
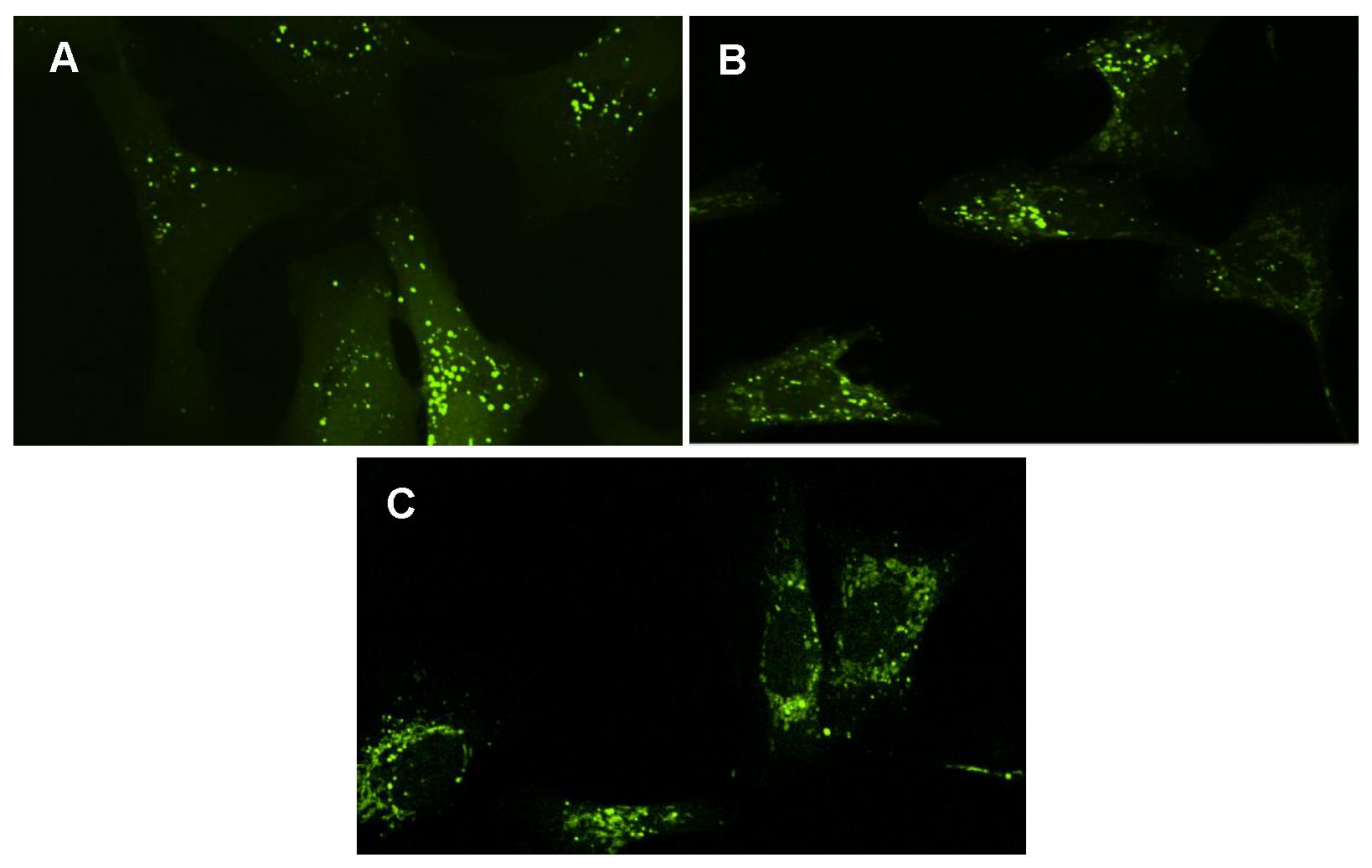
rHSA-p53 and rHSA-PMI are efficiently taken up into SJSA-1 cells. FITC-labeled rHSA (5 μM), FITC-rHSA-P53i (5 μM), and FITC-rHSA-PMI (5 μM) were added to SJSA-1 cells as described in *Methods*. Visualization at 60X magnification revealed efficient uptake of FITC-rHSA (A), FITC-rHSA-P53i (B) and FITC-rHSA-PMI (C) occurred following 24-hour incubation. FITC staining of vesicular cargo suggests significantly greater uptake of rHSA-P53i and rHSA-PMI, compared to rHSA.

While the exact mechanism underlying the uptake of rHSA-P53i and rHSA-PMI is beyond the scope of this paper, we sought to confirm both rHSA fusion proteins are in fact taken up by cells, a critical step to target intracellular proteins. To do this, confocal microscopy was employed to visualize the extent of internalization of FITC-labeled rHSA fusion proteins by SJSA-1 cells. Cells were treated with 5 μM FITC-rHSA, FITC-rHSA-P53i or FITC-rHSA-PMI in the presence of 1% FBS for 24 hours. Depicted in Figure 2A-C, all three proteins (rHSA, rHSA-P53i, and rHSA-PMI) were taken up into SJSA-1 cells. In comparison to rHSA, treatment with rHSA-P53i and rHSA-PMI resulted in robust intracellular vesicle formation and distribution. 

### rHSA-P53i and rHSA-PMI bind both MDM2 and MDMX

**Figure 3:**
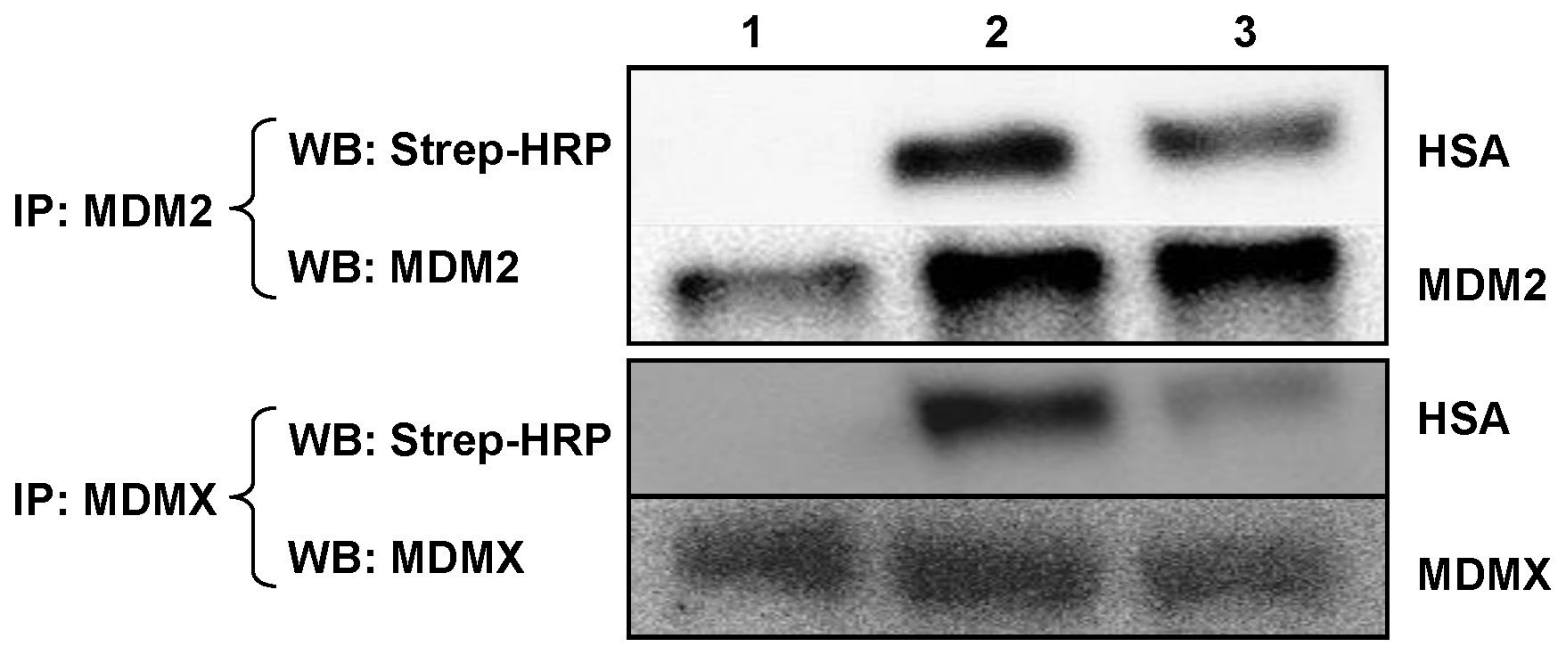
rHSA-P53i and rHSA-PMI bind to MDM2 and MDMX. To detect the interaction between MDM2/MDMX and rHSA fusion proteins, 4 µg each of biotinylated rHSA (lane 1), rHSA-P53i (lane 2), or rHSA-PMI (lane 3) were added to 200 µg of SJSA-1 whole cell lysate. MDM2 or MDMX antibody was added to the lysate followed by pulling down MDM2/MDMX and rHSA complexes using Protein A/G (1:1) resins. Samples were then analyzed by SDS-PAGE and Western blotting using MDM2, MDMX, and Streptavidin-HRP (Strep-HRP) antibodies.

Considerable evidence reveals that disruption of p53-MDM2 interaction can lead to accumulation of p53 and restoration of its tumor-suppressive function [9,10]. Both rHSA-P53i and rHSA-PMI were designed to elicit inhibitory activity against MDM2 and its homolog, MDMX [8,32]. To confirm both rHSA fusion proteins possess MDM2/MDMX binding ability, SJSA-1 whole cell lysate was incubated in the presence of biotin-rHSA, biotin-rHSA-P53i or biotin-rHSA-PMI. Proteins were then pulled down using anti-MDM2 or anti-MDMX antibodies and followed by Western blotting using streptavidin-HRP to detect biotinylated rHSA bound to MDM2/MDMX. Reciprocal detection was performed using streptavidin resins to pull down biotinylated rHSA protein followed by Western blotting using anti-MDM2 or anti-MDMX antibodies (data not shown). As depicted in Figure 3, both rHSA-P53i and rHSA-PMI co-immunoprecipitated with MDM2 and MDMX, thus confirming that target protein binding ability was retained following peptide fusion to HSA.

### rHSA-P53i and rHSA-PMI promote cytotoxicity via caspase activation

**Figure 4:**
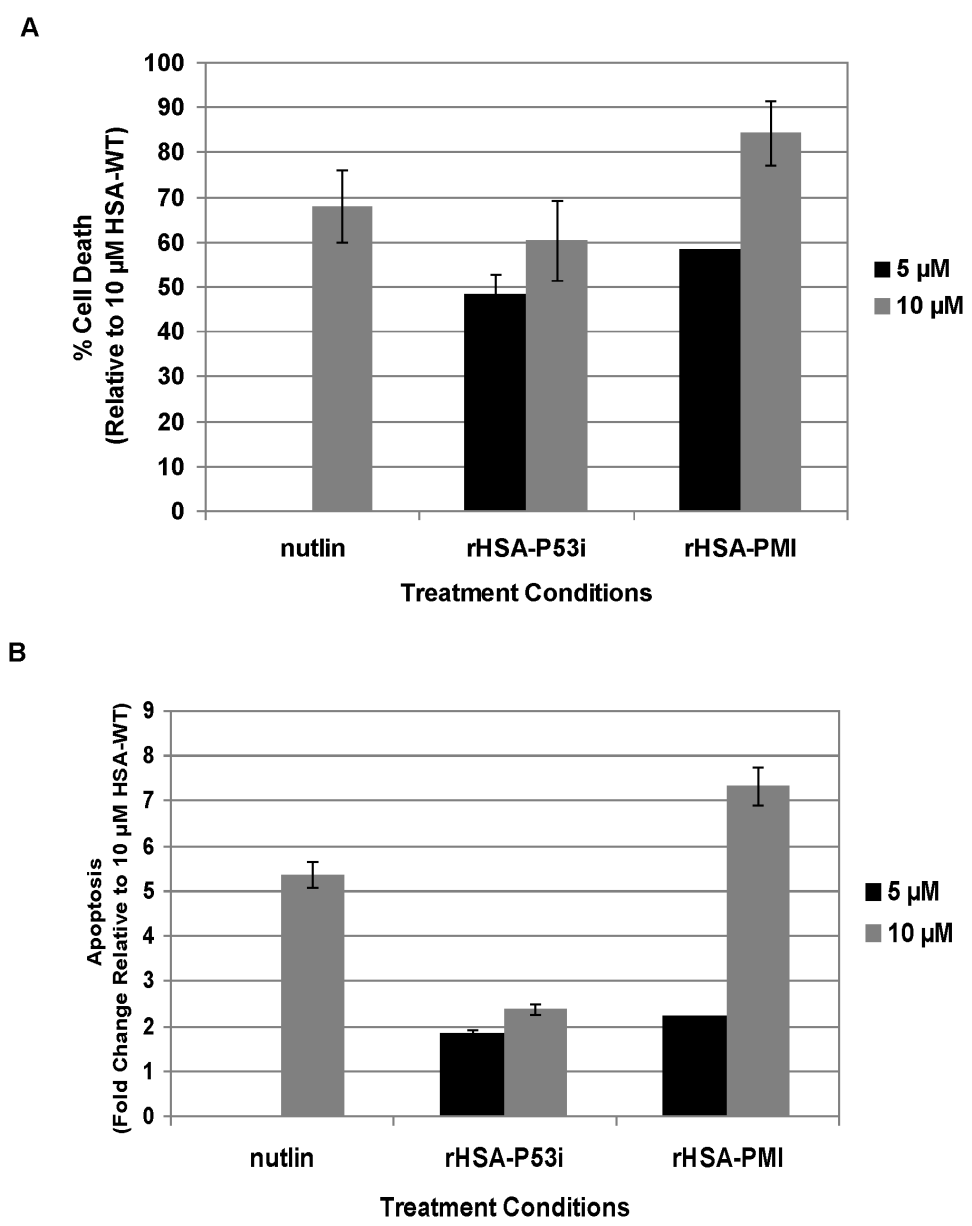
rHSA-P53i and rHSA-PMI promote cytotoxicity in SJSA-1 cells via caspase activation. rHSA fusion proteins, as well as nutlin (to serve as a p53-MDM2 antagonist control) were added at the indicated concentrations and allowed to incubate for 24 hrs. A). Cytotoxicities were measured by CyQuant Assay and normalized according to 10 μM rHSA-treated cells. B). Caspase activation was quantitated using the Homogeneous Caspase Assay as described in *Methods* and normalized according to untreated cells. Results are displayed as percent cell death (A) or fold change (B) relative to 10 μM rHSA-treated wells. Data are representative of 3 independent experiments performed in triplicate. Error bars indicate ± SD.

Adequate levels of p53 are necessary to mediate the cytotoxic effects from chemotherapy or radiation treatment and restoration of its activity has been found to promote tumor regression in mice [4,33]. The small molecule p53-MDM2 antagonists, nutlins, have provided a proof-of-concept that competition with endogenous p53 at the hydrophobic binding cavity of MDM2, can result in accumulation of p53 and initiation of apoptosis [9,10]. To determine if rHSA fusion proteins are able to trigger apoptosis via p53 activation, the MDM2-overexpressing cell line, SJSA-1, was incubated with rHSA-P53i, rHSA-PMI or nutlin. Following a 24-hour treatment, cytotoxicity was assessed using the CyQuant Assay. Both rHSA-P53i and rHSA-PMI promoted cytotoxic responses in SJSA-1 cells (approximately 60% and 84% cell death, respectively) (Figure 4A).

p53 functions as a tumor suppressor by promoting the expression of pro-apoptotic proteins capable of triggering apoptosis via caspase activation. To determine if the cytotoxic response observed was indeed occurring as a result of an apoptotic mechanism, SJSA-1 cells were treated as described above and analyzed for caspase activation. Results in Figure 4B reveal an approximate 7-fold increase in caspase activation in rHSA-PMI-treated cells and up to 2-fold increase in all other treatments. These data confirm both rHSA-P53i and rHSA-PMI promoted cytotoxicity in SJSA-1 cells and this response was driven by apoptotic mechanisms.

### rHSA-P53i and rHSA-PMI promote p53 accumulation

**Figure 5:**
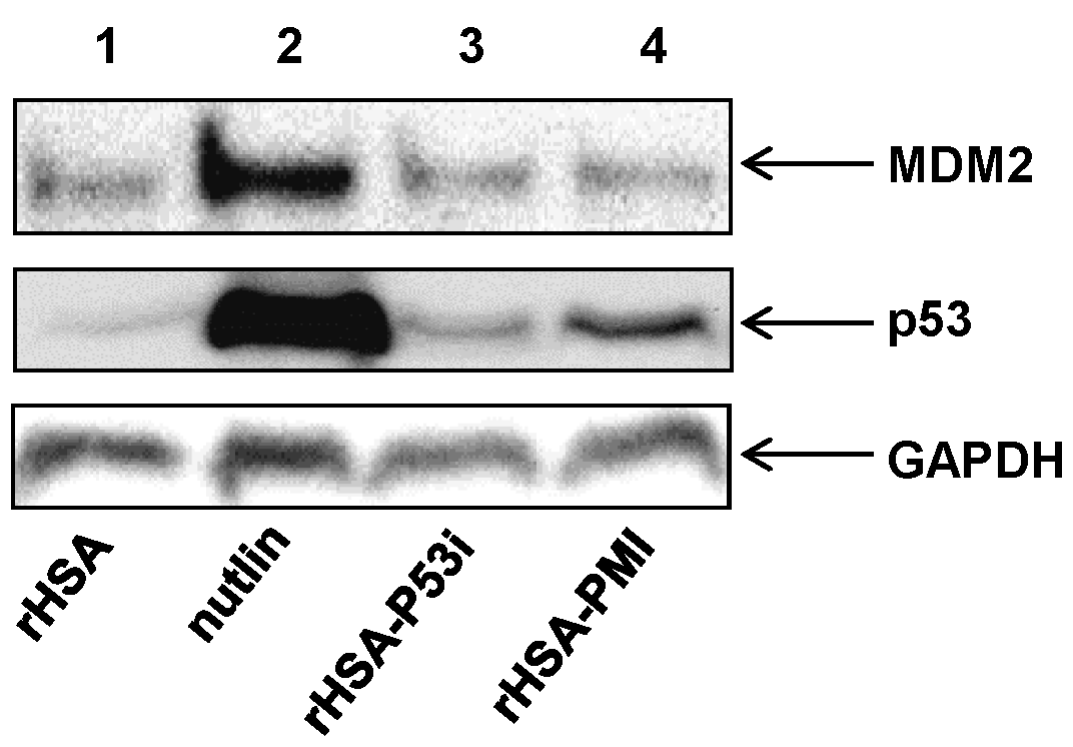
rHSA-P53i and rHSA-PMI induce p53 accumulation, but not MDM2. SJSA-1 cells were plated and allowed to attach overnight. On day 2, culture media with 10 µM rHSA (lane 1), 10 µM nutlin (lane 2), 10 µM rHSA-P53i (lane 3), or 10 µM rHSA-PMI (lane 4) were added to respective wells and allowed to incubate for 24 hrs. Cells were then washed, lysed and immunoblotted for p53 and MDM2. Western blot analysis to detect p53 protein (middle panel) reveals treatment with rHSA-P53i or rHSA-PMI resulted in modest accumulation of p53. Densitometry analysis reveals p53 accumulation following rHSA-P53i and rHSA-PMI treatment is on average, 1.5 and 2.9 orders of magnitude above control wells, respectively. As expected, nutlin-treatment promotes robust p53 accumulation (11.5-fold average increase). However, unlike nutlin, which promotes a 5-fold increase in MDM2 expression, MDM2 protein remained at basal levels following treatment with rHSA-P53i or rHSA-PMI (1.1- and 1.0-fold change, respectively).

p53 functions as a transcription factor for genes involved in mediating key cellular processes such as, DNA repair, cell-cycle arrest, senescence, and apoptosis. In addition, p53 upregulates MDM2 protein expression, via an autoregulatory feedback loop [34–36]. The results in Figure 3 illustrate that rHSA-P53i and rHSA-PMI are capable of disrupting native p53-MDM2 interaction. To extend these studies, we chose to examine the effect of rHSA fusion proteins on p53 and MDM2 protein expression. In Figure 5A, Western blotting was performed to detect both MDM2 and p53 protein levels in SJSA-1 cells after incubation with rHSA-P53i, rHSA-PMI, nutlin, or media alone for 24 hours. As expected, both rHSA-P53i and rHSA-PMI promoted a mild increase in p53, 1.5- and 2.9-fold increases, respectively. Nutlin-treatment promoted robust p53 accumulation (11.5-fold average increase). However, while nutlin displayed a 5-fold average increase in MDM2 protein expression relative to untreated cells; rHSA-P53i and rHSA-PMI did not promote any significant effects on MDM2, resulting in only 1.1- and 1.0-fold changes in protein levels, respectively. Maintenance of lower MDM2 levels following rHSA fusion protein treatment may confer an advantage over nutlin, as upregulation of MDM2 may counterbalance increases in p53 protein and thus, compromise therapeutic efficacy [37]. 

### FA-modified FITC forms a stable complex with rHSA-P53i and rHSA-PMI

FA modification has previously been used to prolong the half-life of small compounds by facilitating non-specific association with serum albumin and lipoproteins [25]. Here we examine the potential of using rHSA fusion proteins to deliver a FA- Drug. Unlike acylated drugs currently in use [22,23], we plan to use an *in vitro* formulation strategy, whereby a FA-Drug is incorporated into rHSA prior to administration. This method ensures uniform and reproducible complex formation, and guarantees each rHSA protein administered will co-deliver both FA-Drugs and a therapeutic peptide. 

**Figure 6:**
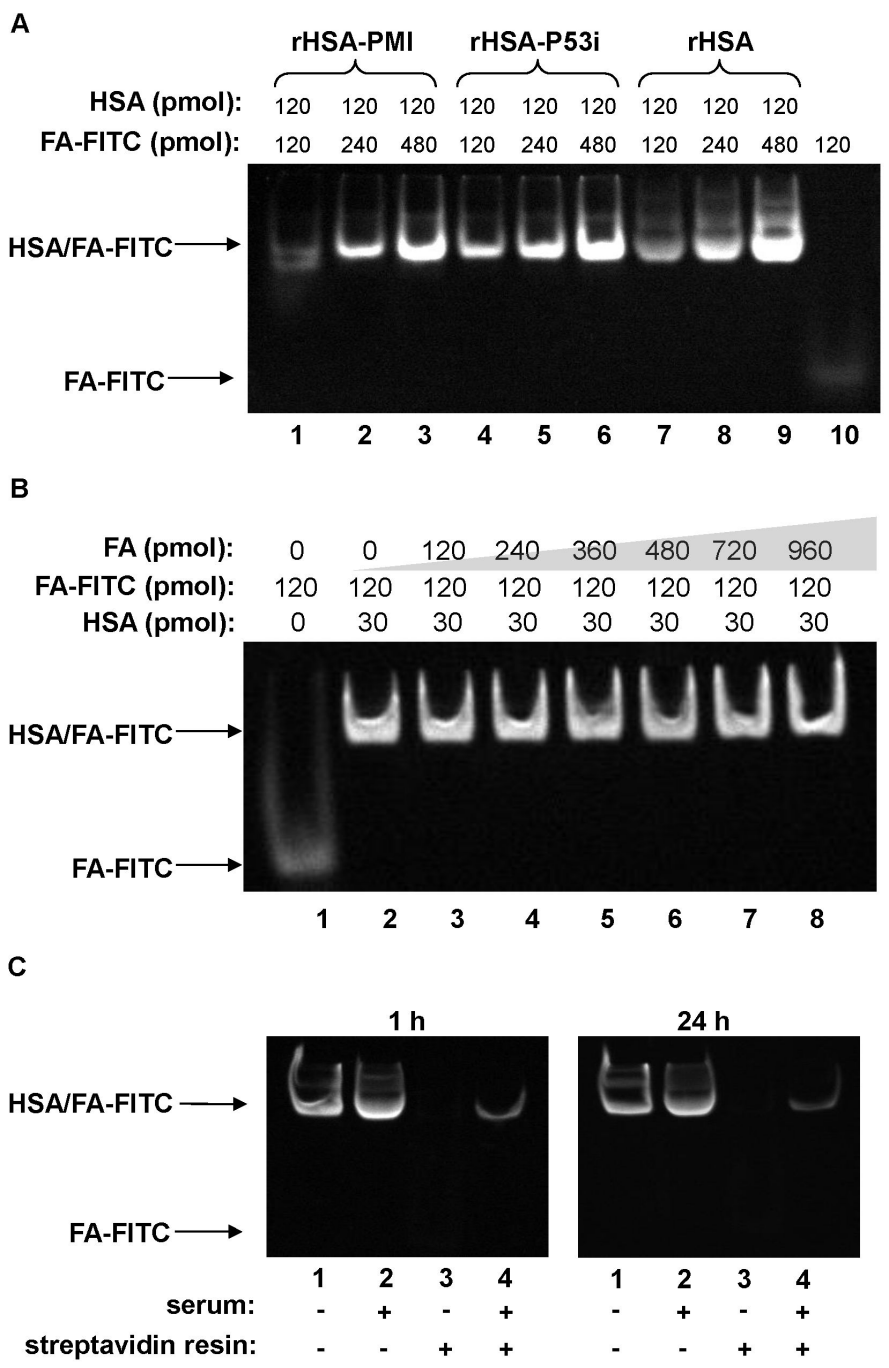
rHSA fusion proteins are able to form stable complexes with FA-FITC. **A**). rHSA-PMI (lane 1-3), rHSA-P53i (lane 4-6) and rHSA (lane 7-9) were incubated at the indicated molar ratios (rHSA:FA-FITC) with FA-FITC (lane 1, 4, and 7 (1:1); lane 2, 5, and 8 (1:2); lane 3, 6, and 9 (1:4); lane 10, FA-FITC only). The upper band in the gel corresponds to the HSA/FA-FITC complex, while the lower band indicates unbound FA-FITC. Incorporation of FA-FITC into rHSA was achieved up to a 1:4 rHSA:FA-FITC molar ratio. **B**). rHSA/FA-FITC complexes were pre-formed at a 1:4 molar ratio (HSA:FA-FITC; 30 pmol:120 pmol) as described in *Methods*. Unlabeled FA was then added at the indicated concentrations to mimic the competition of free FA present under physiological conditions. The minimal dissociation of FA-FITC from pre-formed rHSA/FA-FITC complexes at the 8 times excess concentration of unlabeled FA (lane 8) indicates FA-FITC and rHSA complex was highly stable in the presence of free FA. **C**). Pre-formed biotin-rHSA and FA-FITC complexes (biotin-rHSA:FA-FITC; 1:2) were incubated with PBS (Lane 1 and 3) and 10% serum (lane 2 and 4) for 1 and 24 hours. Lanes 1 and 2 represent total FA-FITC incorporation into rHSA without and with 10% FBS, respectively, prior to the addition of streptavidin resins. Lane 3 (with PBS only) and lane 4 (with 10% FBS) correspond to the supernatants of samples after incubation with streptavidin resins and pulling down rHSA/FA-FITC complexes. The absence of rHSA/FA-FITC complexes in lane 3 indicates that all biotin-rHSA/FA-FITC complexes (in PBS) were efficiently pulled down. Any FA-FITC present in lane 4 would imply the displacement of rHSA-bound FA-FITC by serum components. The presence of only a weak band in lane 4 indicates the majority of FA-FITC remained bound to rHSA (pulled down by streptavidin resins). Quantitation of the amount of FA-FITC in lane 4 (Image J, *NIH*) revealed approximately 15% and 17% of FA-FITC was removed from biotin-rHSA in the presence of serum following 1 and 24 hour incubations, respectively.

In this study, we examined the extent of incorporation and stability of pre-loaded FA using FA-FITC. Recombinant HSA/FA-FITC complex formation was detected using a gel shift assay as described in *Methods*. Complete incorporation of FA-FITC was achieved up to a 1:4 ratio of rHSA:FA-FITC. Notably, the degree of FA binding was similar among rHSA, rHSA-P53i, and rHSA-PMI (Figure 6A). This implies rHSA fusion proteins folded properly and the C-terminal peptide fusion did not alter FA binding ability. As native albumin and free FA will be present under physiological conditions, we designed experiments to mimic an *in vivo* setting in order to assess the overall stability of rHSA/FA complexes. These FA competition assays included: 1) determination of the extent of exchange of HSA-bound FA with excess free FA and 2) assessment as to whether or not incubation in the presence of serum would displace FA from pre-formed rHSA/FA complexes. Our data indicate that rHSA/FA-FITC complexes (formed at 1:4 molar ratio; rHSA:FA-FITC) were stable in the presence of unlabeled FA, up to a 1:8 molar ratio (rHSA-associated FA-FITC:unlabeled FA) (Figure 6B). Next, we examined the extent of exchange of rHSA-bound FA-FITC with lipoproteins and albumin present in serum. To do this, pre-formed biotinylated rHSA/FA-FITC complexes were divided into four separate reaction mixtures. Each reaction condition corresponds to the lane assignments (lanes 1-4), as depicted in Figure 6C. Samples 2 and 4 received 10% serum, while samples 1 and 3 received the same volume of PBS. Each mixture was then allowed to incubate for up to 24 hours at 37°C. To determine the degree of dissociation of FA-FITC from pre-formed rHSA/FA-FITC complexes into serum proteins, streptavidin-conjugated resins were added to samples 3 and 4. This allowed biotin-rHSA/FA-FITC to be pulled down and the supernatants were analyzed for the presence of FA-FITC by gel shift assay. The same aliquots were taken from samples 1 and 2 to serve as positive controls for the determination of total HSA-bound FA-FITC present in each sample prior to the addition of streptavidin-conjugated resins. Any appearance of FA-FITC in lanes 3 and 4 represented the amount of FA-FITC that dissociated from pre-formed rHSA/FA-FITC complexes. A comparison between lane 1 (total rHSA-bound FA-FITC) and lane 4 (serum-associated FA-FITC) indicates the degree of FA-FITC displacement from rHSA into serum proteins. Approximately 86% of FA-FITC remained bound to rHSA following a 24-hour incubation, demonstrating only minimal exchange of FA-FITC occurred between pre-formed rHSA/FA-FITC and albumin or lipoproteins in serum. 

### FA-FITC and rHSA fusion protein complexes are able to transport FA-FITC and promote cytotoxicity

Acylated drugs currently approved for clinical use rely on HSA to enhance solubility and mediate transport to locations within the vicinity of target tissue. Studies have shown that while no gross structural disorganization occurs upon FA incorporation into HSA, FAs have been observed to stabilize the protein against denaturation during clinical applications, indicating some subtle structural changes may occur [38]. It has been demonstrated that FA-FITC and HSA form a stable complex. However, it is uncertain whether the complex interferes with the cellular uptake of FA-FITC or the cytotoxic activity of rHSA fusion proteins. 

**Figure 7:**
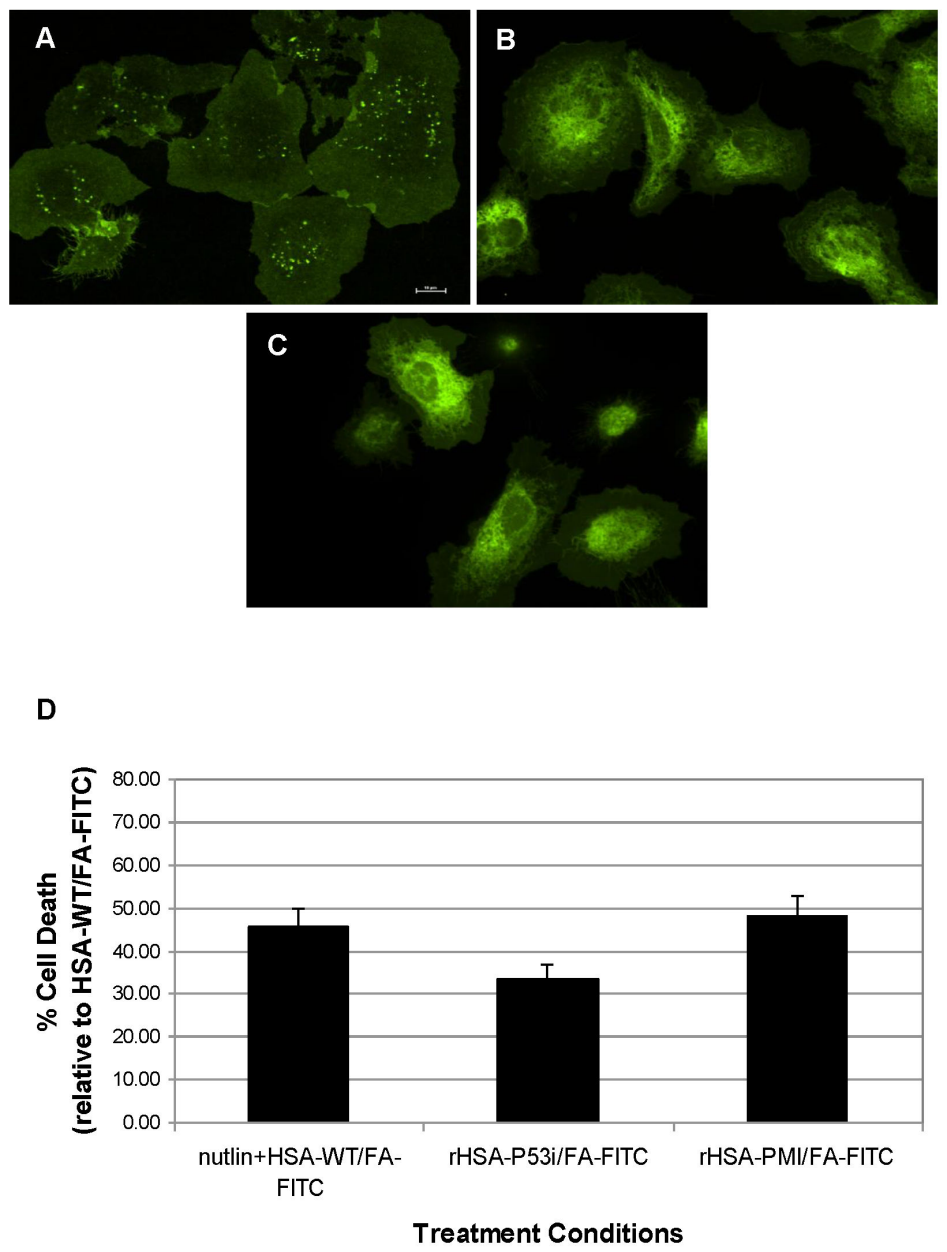
rHSA/FA-FITC complexes retain internalization and cytotoxic activity. **A**). FITC-labeled rHSA (5 μM), **B**). rHSA/FA-FITC (5 μM/10 μM), and **C**). FA-FITC (10 μM) were added to SJSA-1 cells as described in *Methods*. Visualization at 60X magnification revealed efficient uptake of FITC-rHSA, rHSA/FA-FITC and FA-FITC occurred following 24-hour incubation. The extent of FITC staining observed in rHSA/FA-FITC-treated cells is similar to that of FA-FITC treatment alone indicating FA-FITC modification does not impair internalization of rHSA. **D**). rHSA/FA-FITC or rHSA/FA-FITC fusion protein complexes were added at a 1:2 molar ratio (rHSA:FA-FITC; 5 µM:10 µM) to SJSA-1 cells and allowed to incubate for 24 hrs. Nutlin (5 μM) was added in the presence of rHSA/FA-FITC to serve as a positive control. Cytotoxicities were measured by CyQuant Assay as described in *Methods*. Results are displayed as percent cell death relative to 5 μM rHSA/FA-FITC-treated wells. Data are representative of an experiment performed in triplicate. Error bars indicate ± SD.

Confocal microscopy was employed to examine internalization of rHSA/FA-FITC complexes as described in *Methods*. Prior to imaging, SJSA-1 cells were incubated with FITC-labeled rHSA, pre-formed rHSA/FA-FITC complexes, or FA-FITC alone (Figure 7A-C, respectively). As illustrated in Figure 6, we have determined that rHSA/FA-FITC complexes are highly stable even in the presence of 10% serum. The results in Figure 7 demonstrate uptake of FA-FITC, pre-formulated with rHSA (Figure 7B), is similar to that of FA-FITC directly added to the culture medium (Figure 7C). The diffuse FITC staining in Figure 7B indicates HSA/FA-FITC complexes do not interfere with uptake of FA-FITC or affect intracellular distribution of FA-FITC. 

To assess the effect of FA-FITC on rHSA fusion proteins, rHSA/FA-FITC, rHSA-P53i/FA-FITC, and rHSA-PMI/FA-FITC complexes were formed at a molar ratio of 1:2 (rHSA:FA-FITC; 5 μM:10 μM). These complexes, as well as a positive control containing nutlin plus rHSA/FA-FITC, were added to SJSA-1 cells and allowed to incubate for 24 hours. The results in Figure 7D reveal the cytotoxic effects of both rHSA-P53i and rHSA-PMI complexed with FA-FITC. The cytotoxicity associated with rHSA-P53i/FA-FITC and rHSA-PMI/FA-FITC are comparable to that of 5 μM rHSA-P53i and rHSA-PMI (Figure 4A).

## Discussion

**Figure 8:**
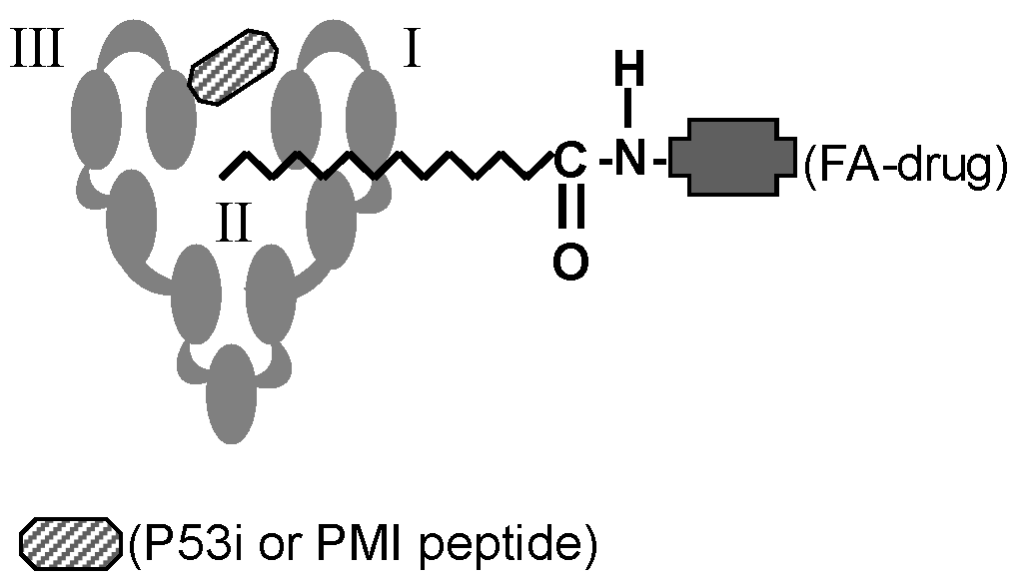
Schematic diagram of rHSA-mediated co-delivery technology. Recombinant HSA-delivery complexes were conceived as a co-delivery technology in that 1) therapeutic peptides can be fused to the C-terminal of HSA for both extracellular and intracellular targeting and 2) FA-Drugs can form stable complexes with rHSA fusion proteins to promote synergistic therapeutic efficacy.

The advent of small peptide therapeutic agents has resulted in the ability to enhance target specificity and blunt toxicity compared to small molecule drugs. Despite these advantages, serum instability and rapid renal clearance have plagued their widespread usage. Here we explore the feasibility of integrating a therapeutic peptide into HSA as an efficient peptide delivery method to target intracellular proteins, circumvent proteolytic degradation *in vivo*, and translate into enhanced serum stability and improved therapeutic efficacy. In addition, this delivery technology has been designed to exploit the intrinsic fatty acid transportation properties of HSA to allow it to co-deliver both a FA-Drug and an intracellular-targeting peptide (e.g. P53i or PMI) to elicit a synergistic therapeutic response (Figure 8).

HSA possesses three homologous domains. Based on physicochemical studies, HSA is a highly flexible protein that is capable of changing its molecular shape under different conditions. The flexibility is partially due to the relative motions of its domain structures. In particular, the two alpha helices in the C-terminal of domain III have minimal interaction with other parts of the protein [16]. The C-terminal is thus the logical location for sequence fusion of therapeutic peptides. The data presented here, using a p53 reactivation model, support our hypothesis that rHSA can be genetically engineered to deliver a therapeutic peptide to an intracellular target and serve as a carrier for FA-modified small molecule drugs. Wild type p53 (P53i) and PMI peptides were fused into HSA by genetic cloning, expressed in a *Pichia pastoris* yeast system, and purified (Figure 1). Intracellular uptake of rHSA-P53i and rHSA-PMI by MDM2-overexpressing SJSA-1 cells was confirmed by confocal microscopy (Figure 2A-C). Furthermore, co-immunoprecipitation assays revealed rHSA fusion proteins were capable of occupying the hydrophobic binding pocket of both MDM2 and MDMX, thus preventing native p53 degradation (Figure 3). These actions resulted in the accumulation of p53 and subsequently, apoptosis (Figures 4 and 5). Lastly, rHSA fusion proteins retained stable FA-binding ability, a critical factor for their eventual application as a carrier for FA-Drugs (Figure 6A). Once forming a complex, rHSA and FA-modified molecules remained stable even in the presence of excess competing free FA (Figure 6B), as well as serum (Figure 6C). 

Improving serum half-life and retaining target protein binding ability is only one of the hurdles that must be overcome for a carrier to successfully deliver functional peptides to a cancer cell. In addition, a drug complex must reach the tumor microenvironment and intracellular uptake must occur to allow for target protein interaction and subsequent therapeutic effect. An important feature of HSA is its ability to cross vascular endothelial cells through albumin-mediated transcytosis and accumulate in the interstitial space of tumor tissues, a process known as the enhanced permeability and retention (EPR) effect [39]. Intracellular uptake of HSA also occurs under conditions of cellular stress, where it serves as a vital source of amino acids. For instance, it has been reported that tumor cells often have an increased rate of HSA uptake [21]. While the precise mode of cellular entry is not entirely clear, studies performed to examine the cellular uptake of Abraxane have revealed that transcytosis is initiated upon binding of HSA to a cell surface glycoprotein (gp60) receptor (albondin), as well as binding of HSA to SPARC (secreted protein acid and rich in cysteine) [40]. Apart from receptor-mediated uptake, fluid phase endocytosis is also suggested to play a role [18]. While future studies will be needed to determine the precise mechanism of rHSA-P53i and rHSA-PMI intracellular uptake, the studies presented here were designed to confirm that rHSA fusion proteins can be efficiently taken up by cancer cells.

To examine cellular uptake, rHSA, rHSA-P53i and rHSA-PMI were fluorescently labeled with FITC. Studies were also performed using FA-FITC and rHSA complexes to examine whether or not this formulation interferes with FA-FITC internalization or rHSA fusion protein activity. Following 24-hour incubation with SJSA-1 cells, confocal microscopy was performed to determine the extent of rHSA fusion protein and rHSA/FA-FITC uptake. Abundant FITC-staining within intracellular vesicles, as depicted in Figure 2, indicates FITC-labeled rHSA-P53i, rHSA-PMI, and rHSA were readily taken up into cells. Interestingly, the degree of internalization was most efficient in cells exposed to rHSA-P53i and rHSA-PMI. Figure 7B confirms FA-FITC, of rHSA/FA-FITC complexes, are also effectively delivered intracellularly based on the extensive FITC staining pattern, which is similar to that of FA-FITC treatment alone. 

Although the neonatal Fc receptor (FcRn) may contribute to albumin internalization, the exact mechanism underlying the increased uptake of rHSA fusion proteins relative to rHSA has yet to be determined. FcRn is a major histocompatibility class I (MHCI) molecule involved in the recycling of both IgG and HSA. It prevents intracellular degradation of protein and prolongs its serum half-life. Importantly, IgG and HSA proteins that do not bind FcRn are retained within the cell and eventually processed into lysosomes for proteolytic degradation. Work performed by Andersen et al. demonstrated that an intact domain III, which contains the C-terminal of HSA, is crucial for FcRn binding and subsequent exporting back to the cell surface [41]. Future studies will be necessary to determine if the C-terminal modification of HSA interferes with FcRn binding, thus promoting intracellular retention of rHSA fusion proteins. 

The overall goal of this technology is to efficiently deliver a therapeutic peptide and potentially, for co-delivery of FA-Drugs to induce a synergistic response. Thus, we next designed experiments to examine the cytotoxic effect of rHSA-P53i and rHSA-PMI. Our data reveal rHSA-P53i and rHSA-PMI, as well as rHSA-P53i/FA-FITC and rHSA-PMI/FA-FITC complexes, caused significant cytotoxicity in SJSA-1 cells (Figure 4A and 7D, respectively). In addition, robust caspase activation was triggered following rHSA fusion protein treatment. This implies toxicity was related to apoptotic mechanisms (Figure 4B). 

To further elucidate the mechanisms underlying the cytotoxic effects of rHSA-P53i and rHSA-PMI, Western blots were performed to examine changes in p53 and MDM2. It has been reported that disruption of p53-MDM2 binding can prevent the sequestration and subsequent ubiquitination of p53 by MDM2 and result in accumulation and reactivation of p53. In line with this mechanism, we observed an increase in p53 protein following a 24-hour incubation with rHSA-P53i, rHSA-PMI or nutlin. Unlike nutlin, treatment with rHSA-P53i or rHSA-PMI did not cause an increase in MDM2. 

The consequences of p53 activation are highly complex and can be different depending on a number of factors, such as differences in stimuli, external environment or cellular milieu. p53-dependent cellular outcomes are dictated by a myriad of transcriptional targets. It has been shown that p53 transcription stimulates the synthesis of MDM2. However, MDM2 can inhibit the transcriptional activity of p53 by binding to its transactivation domain. Furthermore, MDM2 can regulate the degradation of p53 by acting as a shuttle to transport p53 out of the nucleus into the cytosol. Thus, p53 and MDM2 form an autoregulatory feedback loop [34–36]. The dynamic regulatory pathway of MDM2 makes it hard to predict the protein expression outcome caused by p53 activation. In a closed system, it could be assumed that p53 accumulation would lead to an increase in MDM2 protein expression. However, in a cellular context, one must consider that a number of other factors can affect MDM2 stability and activity. As depicted in Figure 5, an increase in p53 protein expression following treatment with rHSA-P53i and rHSA-PMI was observed, while MDM2 remained at basal levels. These results are in contrast to previous studies of MDM2 small molecule antagonists, which observed a concomitant increase in MDM2 upon p53 accumulation [10]. This inconsistency poses an important question: what levels of MDM2 inhibition and p53 activity are required to invoke a beneficial therapeutic effect? To answer this question, work performed by Mendrysa et al. using mouse models with a hypomorphic allele of MDM2, found that even small reductions in MDM2 levels were sufficient to cause a mild p53 response [42]. Based on these studies, our data may suggest p53 activation was beneath the threshold required for promoting p53-mediated transcription of MDM2. A second scenario may also exist in which liberated p53, at different concentrations, triggers transcription of a different subset of genes involved in p53-mediated apoptosis that does not include MDM2. Lastly, we have considered a transcription-independent apoptotic mechanism mediated by cytoplasmic p53 [43]. It was shown that p53-dependent apoptosis still occurred in the presence of transcriptional or translational inhibitors. Furthermore, p53 mutants lacking transcriptional activity retained the ability to trigger apoptotic function. It is possible rHSA-P53i and rHSA-PMI interfere with the transportation of cytoplasmic p53 into the nucleus. Clearly, further studies will be needed to determine whether or not a transcriptional-independent or -dependent apoptotic pathway underlies rHSA fusion protein cytotoxicity, as well as the exact mechanisms underlying the maintenance of MDM2 levels following rHSA fusion protein treatment.

We further explored the potential of using rHSA-P53i or rHSA-PMI as a vehicle to co-deliver a FA-Drug as well as a therapeutic peptide. For ease of quantitation and detection, FITC was chosen as the model molecule to test the feasibility and stability of this co-delivery technology. Recombinant HSA-mediated delivery of FA-Drugs offers a number of advantages over traditional drug delivery methods. These include: 1) HSA association improves solubility of FA-Drugs as well as extremely hydrophobic drugs, 2) formation of FA-Drug and rHSA complex occurs naturally upon incubation and does not require an elaborate chemical reaction, 3) the non-covalent nature of FA-Drug and rHSA negates the need for protein degradation as in polymer/protein-drug conjugates, and 4) drug absorption could also be enhanced due to increased hydrophobicity of the conjugates and HSA-mediated uptake. Generally, LCFAs dissociate from HSA, translocate across the cell membrane, and then reach the mitochondrial membrane and other intracellular locations. The translocation of LCFA across the cell membrane may go through two co-existing mechanisms: simple diffusion and saturable receptor-mediated processes. If a FA-Drug follows the route of LCFA, it may reach the cytoplasmic target through 1) FA transporter-mediated translocation or 2) passive diffusion facilitated by the FA lipophilic alkyl chain. If HSA is involved in the transportation, FA-Drug may translocate across the cell membrane through 1) HSA binding protein-mediated endocytosis or 2) increased HSA uptake in tumor cells [21,40]. Thus, multiple uptake pathways may lead to more efficient drug absorption.

As formulation of rHSA/FA-Drug will be performed *in vitro*, assessing the stability of this complex under simulated *in vivo* conditions was necessary to determine whether significant displacement of FA-Drug from rHSA complexes will occur in the presence of free fatty acids or serum. The work presented here reveals minimal exchange of FA-FITC took place even in the presence of 8 times excess of free FA (Figure 6B). In addition, pre-formulated biotin-rHSA/FA-FITC complexes remained stable for up to 24 hours in the presence of 10% serum, a condition designed to mimic an *in vivo* setting (Figure 6C). 

In recent years, many studies have confirmed that blocking p53-MDM2 interaction holds promise in reestablishing the p53 tumor suppressor pathway when wild type p53 is present. This is particularly relevant in terms of treatment, given that certain cancer cells overexpress MDM2 or MDMX, an MDM2 homolog that also binds and sequesters p53. Structural studies of the p53-binding groove within MDM2 led to the development of both small molecule peptide mimetics (such as nutlins) and rationally designed peptide inhibitors. While nutlins have allowed the mechanistic proof-of-concept for disrupting p53-MDM2 binding for cancer therapy, their pharmacological properties have prevented translation into a clinical model. Peptide inhibitors have the advantage of offering a high degree of target specificity, as well as the ability in some cases to bind and inhibit both MDM2 and MDMX. Despite this potency, however, peptide inhibitors have demonstrated only modest effects in *in vitro* cell models, presumably due to poor membrane permeability and structural stability. Here we present a method that may not only overcome the current obstacles associated with peptide drug delivery into cells, but also facilitates the co-transport of small molecule anticancer agents. Although fatty acid modification may enhance the cellular uptake of molecules, future studies may be needed to optimize the FA conjugation linker to promote maximum internalization and cytotoxic activity of small molecule drug candidates. 
